# Clavicular Metastasis as an Initial Presentation of Papillary Thyroid Cancer

**DOI:** 10.1155/2021/6662071

**Published:** 2021-08-26

**Authors:** Russell Fung, Madeline Fasen, Firas Warda, Patrick Natter, Stacey Nedrud, Rui Fernandes, Ahmad Alkhasawneh, Gunjan Y. Gandhi

**Affiliations:** ^1^Division of Endocrinology, University of Florida College of Medicine-Jacksonville, Jacksonville, FL, USA; ^2^Department of Medicine, University of Florida College of Medicine-Jacksonville, Jacksonville, FL, USA; ^3^Department of Radiology, University of Florida College of Medicine-Jacksonville, Jacksonville, FL, USA; ^4^Department of Oral and Maxillofacial Surgery, University of Florida College of Medicine-Jacksonville, Jacksonville, FL, USA; ^5^Department of Pathology and Laboratory Medicine, University of Florida College of Medicine-Jacksonville, Jacksonville, FL, USA

## Abstract

**Objective:**

We present the case of a 44-year-old man with a large neck mass to highlight the unique presentation of papillary thyroid carcinoma (PTC) metastatic to the clavicle.

**Methods:**

We reviewed the medical record for a detailed history and physical examination findings. Our radiology colleagues examined the diagnostic imaging studies performed. The pathology team reviewed the neck mass biopsy and the confirmatory surgical pathology after total resection of the mass.

**Results:**

A 44-year-old man presented with an enlarging neck mass. Initial X-rays revealed a large soft tissue density mass that extended to the midline of the right clavicle. A neck ultrasound established a 5.4 × 3.6 cm mass with increased vascularity and calcification extending from the thyroid. A CT scan noted the extension of the mass into the adjacent sternoclavicular junction with osteolysis of the middle third of the clavicle and the superior aspect of the sternal body. Fine-needle aspiration revealed a thyroid neoplasm with follicular features and positive immunostaining consistent with thyroid carcinoma. The patient underwent a composite resection of the tumor, including a segmental osteotomy of approximately two-thirds of the medial clavicle. The pathology report confirmed PTC with extrathyroidal extension and clavicle involvement (staged pT4a pN0), with further genomic findings showing positive KRAS mutation.

**Conclusion:**

Clavicular metastasis from differentiated thyroid cancer is rare. While the prognosis is generally favorable, various factors, including age greater than 45 years, poor differentiation, follicular thyroid carcinoma, Hurthle cell variant, and extrapulmonary metastasis, have typically been associated with poorer cancer-specific survival.

## 1. Introduction

Thyroid cancer is a curable disease that affects approximately 53,000 patients annually in the United States, with more than 2,000 deaths each year [1]. Papillary thyroid carcinoma (PTC) remains the most prevalent form of thyroid malignancy and is usually associated with a good prognosis [2]. While metastatic spread from the thyroid gland to the bone is uncommon [3], it has a better survival rate (10-year survival rates ranging from 13% to 21%) than that of other primary carcinomas metastatic to the bone [4]. However, prognosis generally depends on age, tumor burden, and the number of bony metastases at diagnosis [5].

The hypothesis of “seed and soil” is widely accepted in describing the pathophysiology of thyroid carcinoma metastasizing to the bone [6]. This hypothesis indicates that cancer cells circulating in the blood (seeds) tend to metastasize to organs (soil), advantageous for their growth. Bone is a large organ with high calcium, growth factors, and a robust blood supply. These factors are released during bone resorption and provide a fertile microenvironment for growth [7]. Axial skeleton red marrow has been involved in approximately 80% of differentiated thyroid carcinoma metastases due to its high blood flow.

Up to 10% of patients with papillary and follicular thyroid carcinomas present with distant metastases [8]. While the lung is considered the most common site of metastases from thyroid malignancy, bone metastases, if seen, are usually observed at skeletal sites such as the humerus, pelvis, radius, and scapula. PTC metastases to the clavicular bone as an initial presentation is a rare and unique finding [9]. We present a case of PTC that was first noticed as a growth on the right base of the neck and later found to have metastasized to the right clavicle.

## 2. Case Report

**Figure 1:**
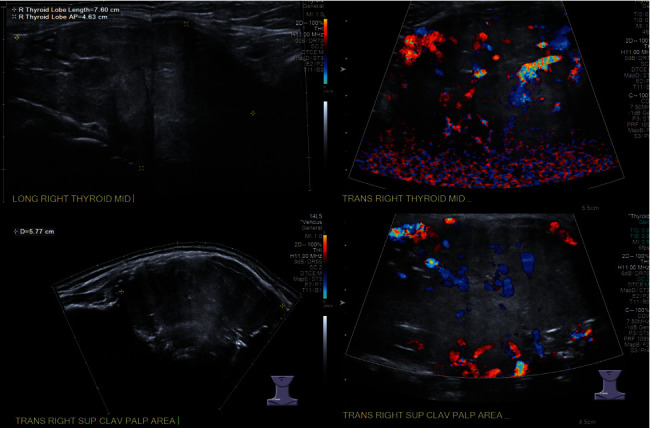
Ultrasound image demonstrating a diffuse mildly heterogeneous enlargement of the right thyroid lobe (a). The associated increased flow within the right thyroid lobe on the color Doppler image (b). A soft tissue mass also seen in the region of palpable concern in the right supraclavicular area, measuring 5.77 cm (c). The associated internal flow in the mass on the color Doppler image (d).

**Figure 2:**
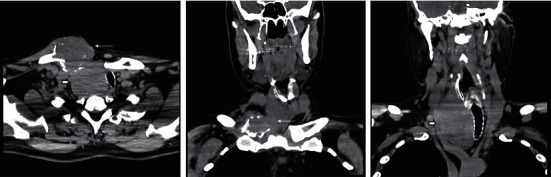
Axial CT image of the soft tissues of the neck (left image) demonstrating a heterogeneous mass within the right clavicular head, which is expansile with associated bony destruction (thin white arrow). There is also mass-like enlargement of the right thyroid lobe (short thick arrow). A coronal CT image of the soft tissues of the neck (middle image) also shows the destructive mass in the right clavicular head with soft tissue component (thin white arrow). A coronal CT image of the neck soft tissues more posterior in position (right image) demonstrates the mass-like enlargement of the right thyroid lobe (short thick arrow).

**Figure 3:**
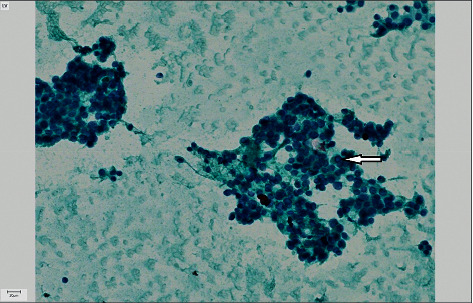
FNA of right neck mass shows cohesive nests of neoplastic thyroid tissue with overlapping nuclei and nuclear pleomorphism. The absence of colloid and macrophages is noted, and a subset of the cells shows nuclear pseudonuclear inclusions (arrow) supporting the diagnosis of papillary thyroid carcinoma (PAP stain, 200x).

A 44-year-old man presented to his primary care provider for a right proximal clavicular mass and enlarging neck for six months that he attributed to an injury from Jujitsu. The patient endorsed mild pain in the area but denied dysphagia, dysphonia, choking, or shortness of breath. The patient reported 20 lbs of weight loss. He denied hypoesthesia or motor deficits. On physical examination, a firm 8 cm mass was fixed to his right clavicle. There was palpable thyromegaly, the right lobe more prominent than the left, but there was no cervical lymphadenopathy and no significant motor or sensory deficits. Initial workup with an X-ray of his right clavicle revealed a large soft tissue density mass extended to the midline of the right proximal clavicle. A subsequent soft tissue neck ultrasound confirmed a 5.4 × 3.6 cm mass extending from the thyroid with features of increased vascularity and calcification extending from the thyroid (Figure 1). CT of the neck showed extension of the mass into the adjacent sternoclavicular junction with osteolysis of the middle third of the clavicle and the superior aspect of the sternum body (Figure 2). Due to suspicion of thyroid malignancy versus osteosarcoma, fine-needle aspiration of the mass was completed that showed a cohesive nest of neoplastic cells, and the nuclei were enlarged with pleomorphism. Focal calcification was noted, but there was no evidence of colloid or macrophages on multiple passes. Immunohistochemical stains were performed on the cell block, and the neoplastic cells were immunoreactive for TTF-1, PAX8, CK7, and thyroglobulin, and they were negative for CK20, calcitonin, synaptophysin, or chromogranin (Figure 3). The overall findings supported the diagnosis of papillary thyroid carcinoma. To assess the extent of the mass for total thyroidectomy, a chest CT scan with contrast confirmed marked enlargement of the right thyroid gland, measuring 5.3 × 7 × 4.9 cm, with invasion into the right proximal clavicle, adjacent strap muscles, and first rib as well as tracheal deviation and extension into the mediastinum.

**Figure 4:**
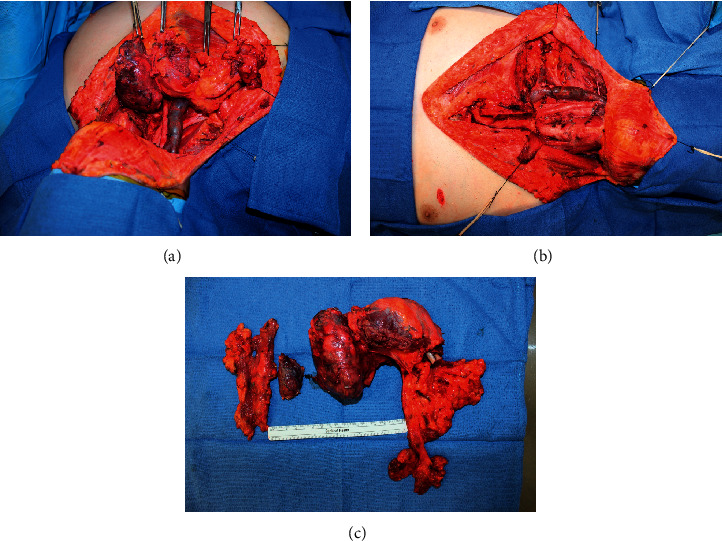
(a), (b) Retracted and removed gross pathology of the PTC mass. (c) Gross pathology of the resected neck mass.

The oral and maxillofacial surgery team performed a composite resection of the tumor, including a segmental osteotomy of approximately two-thirds of the medial clavicle. In continuity with the suprasternal and clavicular portion of the mass, the cardiothoracic surgery team performed a sternotomy to access and resect the substernal component, dissecting it off the first rib and manubrium without a segmental osteotomy. The great vessels were dissected and preserved with dissection of the mass off the superior mediastinum and thoracic inlet. The maxillofacial surgery team continued cephalad to perform a total thyroidectomy. Left levels 2–4, right levels 2–5, and bilateral central compartment selective neck dissections were completed. The mass was then delivered en bloc. The right sternocleidomastoid and strap muscles were sacrificed due to tumor involvement, but all other structures were dissected and preserved, including bilateral recurrent laryngeal nerves and the brachial plexus (Figure 4).

**Figure 5:**
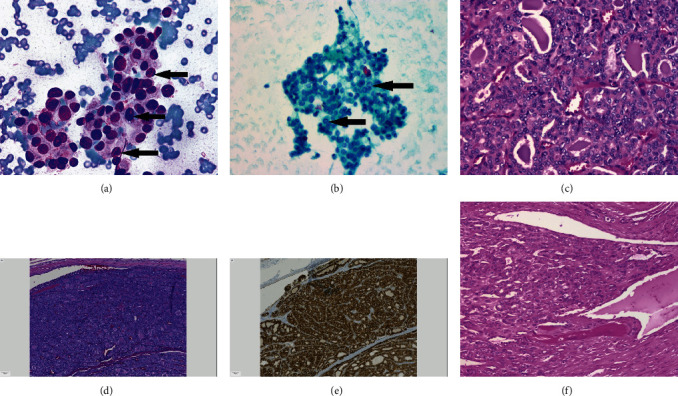
Papillary thyroid carcinoma. (a) Fine-needle aspirate of the right side neck mass showing abundant clusters of neoplastic thyroid tissue, and nuclear grooves (arrow) are present (Diff Quick stain, 200x). (b) Cluster of neoplastic thyroid tissue from the neck mass with focal pseudoinclusions (arrow) in nuclei (PAP stain, 200x). (c) Histologic sections of the right side neck mass showing thyroid carcinoma with papillary nuclear features (nuclear overlap, nuclear grooves, and nuclear clearing), consistent with papillary thyroid carcinoma (H&E stain, 200x). (d) Histologic sections of the thyroid mass in the right lobe showing papillary thyroid carcinoma (H&E stain, 100x). (e) Immunohistochemistry for beta-catenin staining showing no evidence of nuclear staining corresponding to the section of the thyroid mass in D (immunohistochemical stain, 100x). (f) Papillary thyroid carcinoma (follicular variant) involving clavicle (H&E stain, 100x).

Postsurgically, the patient's serum calcium was low 7.9 mg/dL with simultaneously low PTH 9 pg/mL and low 25-hydroxyvitamin D 16.8 ng/mL. Thyroglobulin was markedly high at 15655.0 ng/mL (confirmed on dilution), and thyroglobulin antibody <1.0 IU/mL. Pathological examination of the right thyroid and clavicle showed PTC with extrathyroidal extension and clavicle involvement (staged pT4a pN0) with margins and lymph nodes negative for carcinoma (Figure 5). The tumor showed a predominantly solid pattern with areas of the follicular pattern, but there were no cribriform areas identified. Immunohistochemical stain for beta-catenin showed no evidence of nuclear staining, which is not compatible with the morular pattern of papillary thyroid carcinoma. Next-generation sequencing revealed KRAS Q61 R mutation, and the following mutations were not detected: BRAF, HRAS, TP53, PTEN, TERT, RET, NTRK, and PPARg. The left thyroid showed benign pathology. Due to the perceived need for treatment with radioiodine therapy, the patient was not discharged home on levothyroxine, but was given supplemental calcium and vitamin D with subsequent follow-up with endocrinology.

Postoperatively, the patient's course was complicated by a large expanding left neck hematoma that developed after a fall at home. The patient was readmitted and underwent an emergent hematoma evacuation without further incidence. He was able to follow up with endocrinology with laboratory studies at that time showing serum calcium was 8.6 mg/dL, TSH > 150.00 mIU/L, and free T4 0.2 ng/dL. He was started on calcitriol 0.5 mcg daily), vitamin D 50,000 units weekly, and calcium 1000 mg 4 times daily. Levothyroxine was held at this time with a plan for radioactive iodine treatment. On follow-up, an iodine-123 whole-body scan noted increased uptake over the lower lumbar spine that was suspicious for skeletal metastasis. A positron emission tomography (PET) scan confirmed a high fluorodeoxyglucose (FDG) avid lytic lesion along the posterior element of S2 with a maximum standardized uptake value (SUV) of 13.32. Magnetic resonance imaging (MRI) also revealed an enhancing sacral mass measuring 3.4 cm × 3.3 cm × 2.7 cm at the level of posterior elements of S1 extending to the sacral canal, causing severe sacral canal stenosis. There was a mass effect upon the traversing bilateral S2 nerve roots. These findings were consistent with metastatic disease.

## 3. Discussion

PTC is the most common form of thyroid cancer, accounting for approximately 80% of all thyroid cancers [10]. While bone metastases are rare, they can manifest as a complication of aggressive thyroid carcinoma [3]. Bone has been reported as the second most common metastasis site with well-differentiated thyroid cancer, with follicular carcinoma more prevalent as an etiology (7–28%) than that of PTC origin (1.4–7%) [9, 11]. To this end, our case depicts a unique presentation of thyroidal tissue metastases to the bone due to being confirmed a papillary subtype of well-differentiated thyroid cancer, which typically has a very low propensity for bone metastasis.

Numerous factors contribute to the development of bone metastases. Increased blood flow in bone marrow serves as a powerful mechanism to favor the hematogenous spread of tumor cells [4, 11]. Malignant cells are also known to secrete angiogenesis compounds, which enhance bone resorption, creating an optimal environment for tumors to grow and develop [4]. Thyroid carcinoma is known as an osteolytic tumor. It can enhance the production of IL-1, IL-6, and receptor activator of nuclear factor-kB ligand (RANK-L), which ultimately leads to the resorptive activity of the bone and secondary bone formation as a response [12]. Furthermore, adhesive molecules synthesized by tumor cells allow them to attach to the bone matrix easily. Some studies have shown that bone involvement is more frequent in well-differentiated thyroid cancer than moderately or poorly differentiated thyroid cancer, but this is not conclusive [13, 14]. Typically, cancer cells that exchange biological information with bone environments can establish bone metastases. The “seed and soil” hypothesis is widely accepted [6]. Circulating cancer cells (seeds) have a propensity to metastasize to organs with the microenvironment (soil) advantageous for their growth. The ability of cells to survive, multiply, and recruit a blood supply gives rise to metastases. Bone is a large repository for immobilized growth factors, including transforming growth factors, insulin-like growth factors-I and II (IGF-I and II), fibroblast growth factors, platelet-derived growth factors, bone morphogenetic proteins, and calcium. Released and activated during bone resorption, these factors render the bone fertile for tumor growth [7]. More than 80% of bone metastases from all tumors, including DTC, are in axial skeleton red marrow where blood flow is high (vertebrae, ribs, and hips). In our patient, osseous metastasis to the sacrum is consistent with this finding. Tumor cell adhesive molecules bind the tumor cells to marrow stromal cells and bone matrix, allowing them to grow and produce angiogenic and bone-resorbing factors [11]. Most DTC patients have predominantly osteolytic lesions, with the secondary formation of the bone in response to bone destruction.

Moreover, with respect to metastatic bone lesions, our case is unique, in that it shows primary bone metastasis initially noted to the clavicle. The clavicle is a unique bone from a developmental standpoint. It is known to be the first bone to ossify in the embryo while continuing its ossification process until 18–20 years of age. However, the clavicle's scarce red marrow and scant vascularity make it a rare location for malignancy [15]. As a result, metastatic involvement of the clavicle becomes an even more rare event (0–15% of all known clavicular lesions) with little known about its management [9]. Most presentations of clavicular lesions are reported in small case studies with a broad spectrum of presentations [16]. Regarding the lesion location, the medial third of the clavicular is the most commonly affected location in the clavicular bone, with 60% of the lesions localized to this area [15]. In our case, the lesion encapsulated the medial third of the clavicle, consistent with the most common location of clavicular lesions.

Previous studies have investigated the various prognostic factors in patients with bone metastases from thyroid cancer. These factors, including age (greater than 45 years), poor differentiation, follicular thyroid carcinoma, and Hurthle cell variant, as well as extrapulmonary metastasis, have generally been associated with poorer cancer-specific survival [17]. A study by Durante et al. showed that the 10-year survival was reduced from 80% to 95% in patients with well-differentiated thyroid carcinoma to 14% in patients older than 40 years of age with macronodular lung metastases or multiple bone metastases [18]. Other studies have reported improved overall survival in patients who had lesions that concentrated radioactive iodine and those who had nonosseous lesions [3]. This same investigation reported bone metastasis in 47% of patients, with vertebra (29%), pelvis (22%), ribs (17%), and femur (11%) being the most common sites of metastasis [3]. In a more recent retrospective study that included 64 patients, improved long-term outcomes were associated with younger age, lower tumor stage, no extrathyroidal extension, and nonspinal metastases [5]. Not only was our patient below the age of 45 but his presentation of thyroid extension was also only limited to the clavicular bone with no known evidence of additional metastases. These factors would give our patient a more favorable outcome for his presentation.

While distant metastases from thyroid cancer can be challenging to identify, several imaging modalities are used in its evaluation. While post-I-131 therapy whole-body scans can be quite sensitive in detecting bone lesions, X-rays and bone scintigraphy are also used to evaluate bone metastases [4, 19]. However, the latter is sometimes only useful when more than half of an involved bone has been destroyed [19]. Also, these same modalities are limited by low specificity when pinpointing thyroid cancer as the primary etiology [4, 20]. An advantage of using bone scintigraphy is that it can detect skeletal metastasis much earlier than plain radiographs. But this approach may be of a limited value when evaluating from a thyroid cancer standpoint due to its osteolytic nature [19]. Furthermore, osteolysis can give rise to secondary bone formation in response to bone destruction and contribute to high false-negative and false-positive rates [20].

The biopsy's role in the diagnosis of bone metastasis depends on its clinical presentation and the site of metastasis. Generally, a bone biopsy is unnecessary for those with uptake localized to bone lesions on either diagnostic or posttherapy I-131 scans and in patients with multiple relapses who have previously undergone a biopsy documenting bone metastasis from thyroid cancer. However, a needle biopsy is recommended in the setting of suspected metastatic disease to the spine or pelvis. Biopsy samples should be examined for histological subtypes by utilizing special stains such as the thyroid transcription factor-1 (TTF-1), thyroglobulin (Tg), cytokeratin, and calcitonin [4]. For fine-needle aspirate of nonthyroidal masses with high suspicion of metastases from a thyroid origin, the detection of thyroglobulin levels in the aspirate sample washout can be utilized [21]. Studies of this diagnostic modality have evaluated lymph nodes of patients with PTC, demonstrating sensitivity and specificity close to 100% [22, 23]. While this method has not been extensively studied in bone metastasis cases of thyroid origin, the value of this technique in these specific cases would likely be similar. In our case, the extrathyroidal spread was limited to the right clavicle. Thus, only a biopsy of the neck mass itself was performed as the diagnostic modality. The diagnosis was later confirmed in surgical pathology as PTC.

## 4. Conclusion

We describe a case of PTC with its initial presentation as a neck mass involving the clavicle. While bone metastases from differentiated thyroid cancer are rare, the clavicular spread becomes an even more unique finding arising from PTC. Bone scintigraphy, X-ray, and fine-needle biopsy are some of the widely utilized methods employed in evaluating bone metastasis in the setting of thyroid malignancy. However, one should consider the benefits and limitations of these modalities. While the prospect of recovery is generally favorable, clinicians should be mindful of the various essential factors that determine prognosis in these cases.

Two specimens were received for pathologic evaluation. The first was a composite resection of the right thyroid with attached tissue, including a separate tumor mass, part of the clavicle, and left side neck dissection. The second specimen was an unremarkable left thyroid gland with attached left side neck dissection. The thyroid mass in the right lobe measured 7 cm in the greatest dimension, and the adjacent tissue mass was 10 cm in the greatest dimension. Both masses showed papillary thyroid carcinoma (solid variant and follicular variant). The adjacent tissue mass was separate from the thyroid gland, and the tissue in between the soft tissue mass and the gland was not involved by carcinoma. The adjacent tissue mass may represent either metastatic papillary carcinoma focus versus papillary thyroid carcinoma in aberrant thyroid tissue. However, there was no evidence of nonneoplastic thyroid tissue surrounding the adjacent tissue mass. Immunohistochemical stain for beta-catenin shows no evidence of nuclear staining, which argues against the possibility of a morular pattern of papillary thyroid carcinoma. Next-generation sequencing revealed KRAS Q61 R mutation, and the following mutations were not detected: BRAF, HRAS, TP53, PTEN, TERT, RET, NTRK, and PPARg.
